# Vaginal cuff perforation during robotic‐assisted mesh recto‐sacrocolpopexy

**DOI:** 10.1002/iju5.12138

**Published:** 2020-02-29

**Authors:** Jacqueline Chavez, Carlos Finsterbusch, Craig Olson, Philippe E Zimmern

**Affiliations:** ^1^ UT Southwestern Medical Center Dallas Texas USA

**Keywords:** complication, minimally invasive surgery, pelvic organ prolapse, sacrocolpopexy, vaginal cuff

## Abstract

**Introduction:**

We report on the management of intraoperative vaginal cuff perforation during robotic‐assisted mesh recto‐sacrocolpopexy for vaginal vault prolapse with defecatory dysfunction.

**Case presentation:**

A 75‐year‐old woman with vaginal bulge and constipation was to undergo a joint robotic mesh recto‐sacrocolpopexy. Intraoperatively, mesh was secured to the left posterior vaginal wall following dissection. Prior to contralateral suture placement, the vaginal cuff split open and exposed an end‐to‐end anastomotic sizer previously inserted in the vagina. Due to subsequent mesh erosion risk, we proceeded with vaginotomy closure with running and interrupted absorbable sutures, removal of mesh, direct suture rectopexy to the promontory, and enterocele defect correction by reapproximating the right and left wings of the peritoneum flaps over the rectum with running sutures. Patient reported satisfactory outcomes after 2 years.

**Conclusion:**

We reviewed our experience with vaginal cuff perforation during robotic‐assisted mesh recto‐sacrocolpopexy prompting enterocele repair and rectopexy without mesh.

**Abbreviations & Acronyms**
EEA: end‐to‐end anastomotic
MSC: mesh sacrocolpopexy
POP‐Q: Pelvic Organ Prolapse Quantification system

**Keynote message**

This case report outlines an unreported complication during a robotic‐assisted mesh recto‐sacrocolpopexy. Intraoperative vaginal cuff perforation led to a change in management without mesh use. Following a change in surgical technique, the patient has had satisfactory outcome without prolapse recurrence at medium‐term follow‐up.

## Introduction

Although MSC is the gold standard treatment for apical prolapse,^1^ intraoperative complications are seldom reported.^2^ In one large review, three complications described included cystotomy, enterotomy, or proctotomy, and ureteral injury.^2^ We present a case of vaginal cuff perforation during robotic‐assisted laparoscopic MSC and rectopexy, and its subsequent successful management with medium‐term follow‐up.

## Case presentation

A 75‐year‐old Caucasian, G3P3 female presented to the urology clinic at a tertiary care center with a chief complaint of vaginal bulge and constipation. Past surgical history included an abdominal hysterectomy and Burch colposuspension in 1990, followed by urethrolysis and vaginal repair of stage 3 anterior compartment prolapse in 2000 for voiding dysfunction and recurrent urinary tract infections. Since then, she denied incontinence or residual voiding complaints. However, her constipation worsened requiring digital manipulation for rectal evacuation.

**Figure 1:**
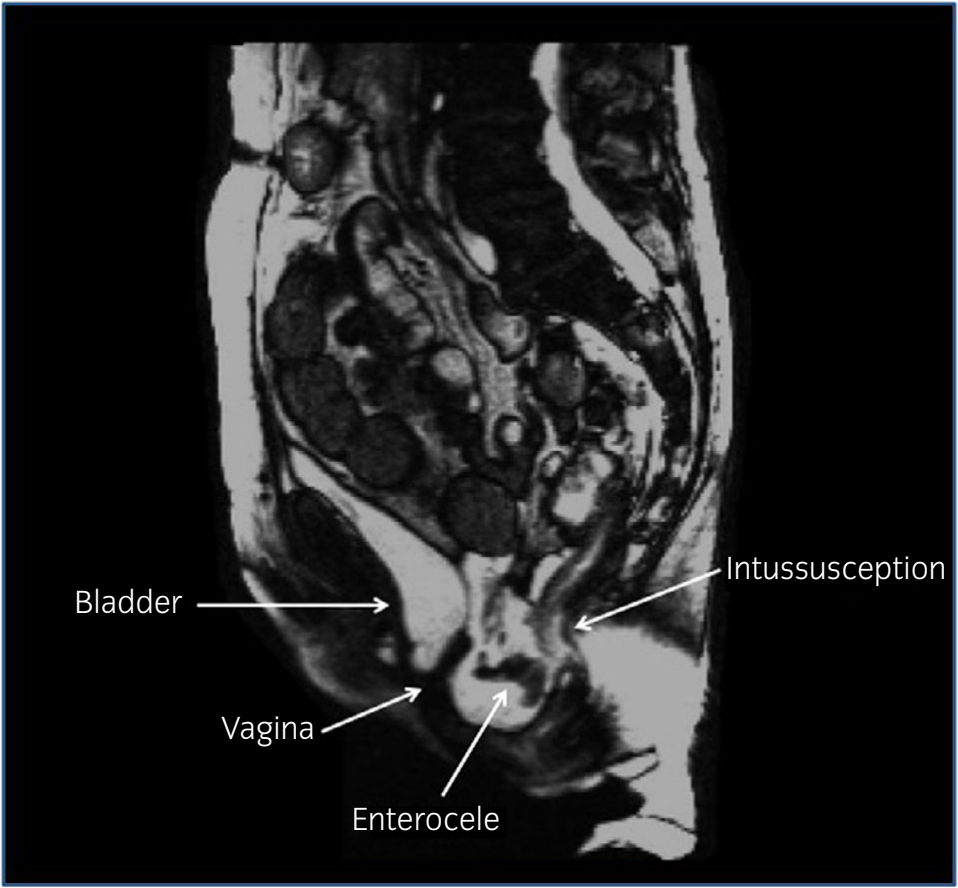
Preoperative magnetic resonance defecography.

Prolapse assessment using the POP‐Q^3^ confirmed a significant posterior compartment bulge (Ap: −2, Bp: 0). Magnetic resonance defecography^4^ reported mild vaginal prolapse (1.7 cm), a moderate cystocele (3.2 cm), a moderate enterocele (5.7 cm), and a moderate rectocele with distal bulge (1.2 cm) along the posterior vaginal wall. Rectal intussusception was also noted with partial thickness mucosal invagination, full‐thickness invagination of the posterior wall of the rectum, and partial anterior wall invagination (Fig. 1). After consultation with the colorectal department, it was decided to proceed with robotic MSC and rectopexy to jointly address her rectocele and enterocele defects.

**Figure 2:**
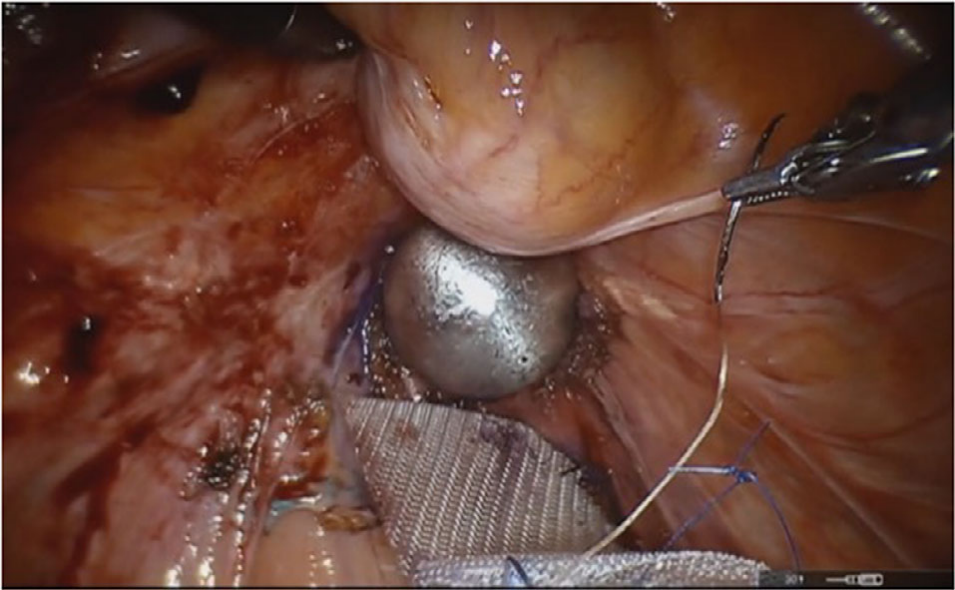
Intraoperative vaginal cuff perforation.

Intraoperatively, a lubricated, round, EEA sizer was inserted in the vagina to facilitate dissection of the vaginal cuff. Following surgical technique we previously described,^5^ the dissection of the enterocele sac, rectovaginal space, and vaginal cuff were performed. The rectum was dissected to allow full mobilization to the promontory area. Once the dissection of the vaginal cuff, rectum, and promontory areas were completed, an Atrium™ mesh was prepared on the back table to fit the vaginal cuff size and then positioned in the pelvis via the assistant port. The mesh was secured to the distal left posterior vaginal wall. While the EEA sizer was oriented upward to expose the posterior vaginal wall in preparation for the placement of the contralateral suture, the vaginal cuff split open and the EEA sizer became fully exposed at the bottom of the pelvic cavity (Fig. 2).

**Figure 3:**
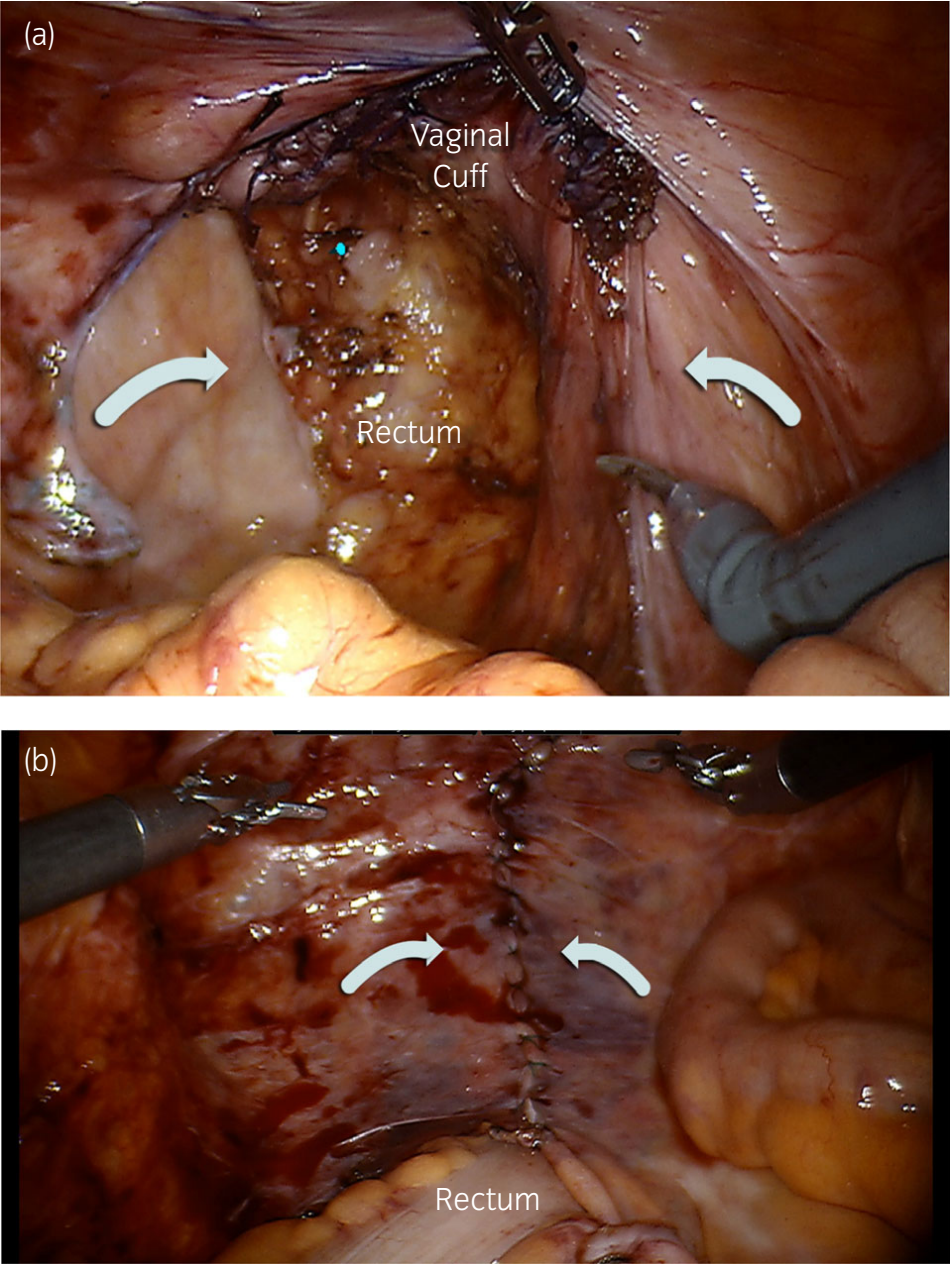
(a) Peritoneal closure via reapproximation of left and right peritoneal flaps. (b) Final peritoneal closure.

Due to the risk of mesh contamination and very thin vaginal tissues, the multidisciplinary team decided to forgo mesh placement. The vaginotomy was closed with running and interrupted absorbable sutures, and the mesh was removed. A direct suture rectopexy to the promontory was performed to tent the rectum up. Next, the enterocele defect (Fig. 3a) was corrected by reapproximating the right and left wings of the peritoneum flaps over the midline and in front of the rectum using running 3–0 V‐Loc sutures (Fig. 3b).

On follow‐up visits, patient confirmed relief of the vaginal bulging sensation with some residual constipation easily managed with stool softeners. At last assessment 2 years after robotic surgery, she still required management of her constipation; however, physical examination had remained unchanged over time and revealed POP‐Q Stage 1 prolapse (Aa: −2, Ba: −2, Ap: −3, Bp: −3, TVL: 8, and C: −7) without changes in bladder function (post‐void residual: 0 mL) or return of her rectal prolapse.

## Discussion

Given the prevalence of POP and the aging of the population, the rates of MSC procedures have increased.^6^ We intended to correct the defecatory dysfunction by placement of mesh secured over the rectum and posterior vaginal wall compartment with upper fixation to the promontory. Following vaginal cuff perforation, suture rectopexy was used, and the enterocele defect was closed with direct peritoneal midline reapproximation over the rectum. Concern for secondary vaginal exposure, possible secondary mesh infection, and alternative techniques available for enterocele repair guided our decision to not use mesh over a closed vaginal cuff.

Management of intraoperative complications varies based on surgeon experience. O'Sullivan and colleagues surveyed 189 urogynecologists regarding approaching intraoperative complications during MSC and found 5% would abandon the procedure following bladder injury and 45% after rectal injury.^7^ A rare event such as vaginal cuff perforation was not addressed, or was it mentioned as an intraoperative complication in a large review of 2178 abdominal MSC cases by Nygaard and colleagues.^2^ Once the vaginal cuff perforation occurred, our case presented similarly to one in which MSC is conducted after a hysterectomy. Mesh exposure to vaginal microbes in cases of concurrent hysterectomy poses a risk of contamination and subsequent mesh infection.^2^ In fact, Culligan and colleagues reported an erosion rate of 27% (3/11) in women who underwent MSC with concomitant total hysterectomy compared to 1.3% (3/234) in MSC alone (*P *<* *0.001).^8^

Regarding surgical technique, suture rectopexy has been described for the treatment of rectal prolapse since 1922^9^ with a 2–9% recurrence rate at medium‐term follow‐up,^10^, ^11^, ^12^, ^13^ which are similar to those reported for mesh rectopexy.^14^ Subjective postoperative constipation symptoms vary. Kessler *et al*. noted a 6% (2/32) prevalence at median follow‐up of 33 (3–78) months,^11^ whereas Novell *et al*. reported 31% (10/32) at median follow‐up of 47 (2–104) months.^13^

Many surgeons employ techniques during POP repair to prevent subsequent enterocele formation, including the Moschcowitz or Nichols techniques. In this instance, a neo‐douglas formation was obtained by approximating over the midline and in front of the rectum the left and right medial borders of the incised peritoneum using resorbable sutures.^15^

To mobilize the vaginal apex during dissection, a round, medium‐sized EEA sizer was selected based on the size of the patient's vagina. Other vaginal mobilization options include malleable blades or sponge sticks. Careful consideration should be taken to ensure that added stress is not placed on the vaginal cuff due to sharp edges or improper sizing. Furthermore, dissection of the peritoneum overlying the vaginal apex to serve as an anchor point to secure the mesh can be challenging in the absence of haptic feedback. This might have been a contributing factor in this case due to dense scar encountered over the vaginal apex from her prior procedures. Experience with cuff dissection could be questioned; however, we have reported our outcomes of abdominal MSC in 29 women from 2000 to 2006^16^ as well as long‐term results with robotic MSC over 56 patients from 2007 to 2012^5^ and thus far had not encountered such an intraoperative event.

## Conclusion

We present our experience with a vaginal cuff perforation during a robotic‐assisted mesh recto‐sacrocolpopexy. This sudden event led to a shift in surgical technique to repair the prolapse defects while avoiding mesh use.

## Conflict of interest

The authors declare no conflict of interest.

## References

[1] Barber MD , Maher C . Apical prolapse. Int. Urogynecol. J. 2013; 24: 1815–33. 24142057 10.1007/s00192-013-2172-1

[2] Nygaard IE , McCreery R , Brubaker L *et al* Abdominal sacrocolpopexy: a comprehensive review. Obstet. Gynecol. 2004; 104: 805–23. 15458906 10.1097/01.AOG.0000139514.90897.07

[3] Madhu C , Swift S , Moloney‐Geany S , Drake MJ . How to use the Pelvic Organ Prolapse Quantification (POP‐Q) system? Neurourol. Urodyn. 2018; 37(S6): S39–43. 30614056 10.1002/nau.23740

[4] Al‐Najar MS , Ghanem AF , AlRyalat SAS , Al‐Ryalat NT , Alhajahjeh SO . The usefulness of MR defecography in the evaluation of pelvic floor dysfunction: our experience using 3T MRI. Abdom. Radiol. 2017; 42: 2219–24. 10.1007/s00261-017-1130-7 28386692

[5] Jong K , Klein T , Zimmern PE . Long‐term outcomes of robotic mesh sacrocolpopexy. J. Robot Surg. 2018; 12: 455–60. 28980173 10.1007/s11701-017-0757-2

[6] Wu JM , Kawasaki A , Hundley AF , Dieter AA , Myers ER , Sung VW . Predicting the number of women who will undergo incontinence and prolapse surgery, 2010 to 2050. Am. J. Obstet. Gynecol. 2011; 205: 230.e1–5. 21600549 10.1016/j.ajog.2011.03.046 PMC3630997

[7] O'Sullivan OE , Matthews CA , O'Reilly BA . Sacrocolpopexy: is there a consistent surgical technique? Int. Urogynecol. J. 2016; 27: 747–50. 26564217 10.1007/s00192-015-2880-9

[8] Culligan PJ , Murphy M , Blackwell L , Hammons G , Graham C , Heit MH . Long‐term success of abdominal sacral colpopexy using synthetic mesh. Am. J. Obstet. Gynecol. 2002;187:1473–80. 12501049 10.1067/mob.2002.129160

[9] Rickert A , Kienle P . Laparoscopic surgery for rectal prolapse and pelvic floor disorders. World J. Gastrointest. Endosc. 2015; 7: 1045–54. 26380050 10.4253/wjge.v7.i12.1045 PMC4564831

[10] Wilson J , Engledow A , Crosbie J , Arulampalam T , Motson R . Laparoscopic nonresectional suture rectopexy in the management of full‐thickness rectal prolapse: substantive retrospective series. Surg. Endosc. 2011; 25: 1062–4. 20835728 10.1007/s00464-010-1316-3

[11] Kessler H , Jerby BL , Milsom JW . Successful treatment of rectal prolapse by laparoscopic suture rectopexy. Surg. Endosc. 1999; 13: 858–61. 10449838 10.1007/s004649901119

[12] Blatchford GJ , Perry RE , Thorson AG , Christensen MA . Rectopexy without resection for rectal prolapse. Am. J. Surg. 1989; 158: 574–6. 2589593 10.1016/0002-9610(89)90196-7

[13] Novell JR , Osborne MJ , Winslet MC , Lewis AA . Prospective randomized trial of Ivalon sponge versus sutured rectopexy for full‐thickness rectal prolapse. Br. J. Surg. 1994; 81: 904–6. 8044618 10.1002/bjs.1800810638

[14] Faucheron JL , Trilling B , Girard E , Sage PY , Barbois S , Reche F . Anterior rectopexy for full‐thickness rectal prolapse: technical and functional results. World J. Gastroenterol. 2015; 21: 5049–55. 25945021 10.3748/wjg.v21.i16.5049 PMC4408480

[15] D'Hoore A , Penninckx F . Laparoscopic ventral recto(colpo)pexy for rectal prolapse: surgical technique and outcome for 109 patients. Surg. Endosc. 2006; 20: 1919–23. 17031741 10.1007/s00464-005-0485-y

[16] Gilleran JP , Zimmern P . Abdominal mesh sacrocolpopexy for recurrent triple‐compartment pelvic organ prolapse. BJU Int. 2009; 103: 1090–4. 19154495 10.1111/j.1464-410X.2008.08296.x

